# The Non-Surgical Treatment of Splanchnoptosis

**Published:** 1917-12

**Authors:** Rolla Camden

**Affiliations:** Parkersburg, W. Va.


### THE NON-SURGICAL TREATMENT OF SPLANCHNOPTOSIS.

**By Rolla Camden, M.D., Parkersburg, W. Va.**

Without going deeply into the etiology, pathology and symptoms and diagnosis of splanchnoptosis, attention is called to the necessity for treatment first along conservative lines of medicinal, personal and mechanical aids in this condition rather than the surgical treatment usually offered.

Normally, the viscera are held in place by their respective supports, and intra-abdominal pressure, these being aided by proper poise of the body.

In splanchnoptosis the poise is bad, with the resulting loss of intra-abdominal pressure and of supporting visceral attachments.

Splanchnoptosis divided into simple and complex: Simple when there are no restraining adhesions and the organs are returnable to their normal positions; complex (extravisceral) when they are retained out of place by adhesions and are not, therefore, returnable to their normal places.

Our endeavor should be to restore viscera to their normal places and maintain them there. This is to be accomplished by personal efforts of the patient by correction of posture, certain abdominal exercises, massage, vibration and hydrotherapy, and medical treatment as indicated after gastric and enterio analyses.

Other means are external or mechanical, such as shoulder braces, abdominal supports, corsets, etc., modified to suit the patient and raising foot of the bed.

Attention is called to the difficulty of obtaining proper and constant support to thin individuals with small abdomens and prominent hip-bones, with suggestions for modifying the usual mechanical aids offered.

